# How selection structures species abundance distributions

**DOI:** 10.1098/rspb.2012.1379

**Published:** 2012-07-11

**Authors:** Anne E. Magurran, Peter A. Henderson

**Affiliations:** 1 School of Biology, University of St Andrews, St Andrews, Fife KY16 8LB, UK; 2 PISCES Conservation Ltd, IRC House, The Square, Pennington, Lymington, Hants SO41 8GN, UK

**Keywords:** biodiversity, predation, estuarine fish, body size, biomass

## Abstract

How do species divide resources to produce the characteristic species abundance distributions seen in nature? One way to resolve this problem is to examine how the biomass (or capacity) of the spatial guilds that combine to produce an abundance distribution is allocated among species. Here we argue that selection on body size varies across guilds occupying spatially distinct habitats. Using an exceptionally well-characterized estuarine fish community, we show that biomass is concentrated in large bodied species in guilds where habitat structure provides protection from predators, but not in those guilds associated with open habitats and where safety in numbers is a mechanism for reducing predation risk. We further demonstrate that while there is temporal turnover in the abundances and identities of species that comprise these guilds, guild rank order is conserved across our 30-year time series. These results demonstrate that ecological communities are not randomly assembled but can be decomposed into guilds where capacity is predictably allocated among species.

## 1. Introduction

Species abundance distributions (SADs) capture the inequality of species abundances that characterize every ecological community [1]. The appreciation that species vary in their commonness and rarity has deep roots in ecology; Darwin [2], for example, drew on this observation when formulating his ideas about natural selection. Despite the ubiquity of these patterns, and the large literature on SADs, we still have an incomplete understanding of the mechanisms that shape species abundances.

To explain SADs, we need to consider two factors. First, it is essential to ask how biomass is allocated among species. This is key, because biomass is directly linked to resource use, particularly where species or individuals differ markedly in body size [3,4]. Second, by partitioning the community into the component functional groups that exploit different parts of the spatial domain [5,6], we can ask how selection influences the distribution of biomass in relation to body size. Body size affects the efficiency with which organisms turn available energy into new biomass [7–9], such that species with larger individuals produce more biomass on a *per capita* basis [7,10]. But body size is also a target of both natural and sexual selection that can offset the increased energetic efficiency of size. Predators, for example, exert strong selection on numerous traits, including body size. Animals that live in open habitats often rely on safety in numbers defences [11,12] which select for biomass to be divided into larger numbers of smaller individuals. In such cases, we predict that larger bodied species will be responsible for a reduced fraction of total biomass. Here we use this two-step approach to make testable predictions about SADs in local communities. We conclude by arguing that SADs emerge when the distributions of biomass in different spatial guilds are summed, and that by taking into account heterogeneity in how selection operates on body size we can make the link with the distributions of numerical abundance typically collected by field workers.

We test our contention that there are predictable differences in the distribution of biomass among spatial guilds using an exceptionally well-documented estuarine fish community that has been sampled monthly for 30 years, and in which the 81 species belong to distinct spatial guilds. These guilds exploit the available habitat in different ways [13] and include open water taxa, and those associated with soft and rocky bottom habitats. They are pelagic, proximo-benthic, hard-benthic, soft-benthic, weed and sheltered shallow guilds (see table 1 for an explanation and examples). The first four of these contain most species (greater than or equal to 13 each) and are the focus of our analysis. In addition, there are a few migratory species that pass through the estuary in modest numbers. The categorization of species into guilds is based on expert knowledge and was done by one of us (P.A.H.) independently of the analysis. Because guilds exploit spatial zones that have not changed through the duration of the study we expect guild rank order to have been maintained through time. Guilds do not differ in trophic level (*F*_1,65_ = 0.29 *p* = 0.59 and see electronic supplementary material, figure S1), a result that reflects the fact that in inshore fish communities large, e.g. basking shark, *Cetorhinus maximus* (which weighs up to 4 000 000 g) and small taxa, e.g. transparent goby, *Aphia minuta* (up to 2 g) can have similar planktonic diets.

**Table 1.** Definitions of the spatial guilds present in this assemblage, with examples of species in each guild and guild size.

spatial guild	definition	examples	no. species
pelagic	open water species not adapted to deal with surfaces	herring, *Clupea harengus*	13
		sprat, *Sprattus sprattus*	
proximo-benthic	species of free swimming fish which tend be found close to structures such as reefs or sand waves	bass, *Dicentrarchus labrax*	14
		whiting, *Merlangius merlangus*	
hard-benthic	fish associated with hard surfaces and which normally rest on or under the seabed, or hidden within crevices	5-bearded rockling, *Ciliata mustela*	14
		conger eel, *Conger conger*	
soft-benthic	as hard benthic but associated with soft sediment	flounder, *Platichthys flesus*	26
		Dover sole, *Solea solea*	
weed	fish associated with seagrass and seaweed	black goby, *Gobius niger*	6
		15-spined stickleback *Spinachia spinachia*	
sheltered shallow	species favouring harbours, lagoons and other inshore, low wave energy habitat.	thick-lipped grey mullet, *Chelon labrosus*	4
		thin-lipped grey mullet, *Liza ramada*	
other	migratory species which are either catadromous or anadromous and pass through the estuary	Atlantic salmon *Salmo salar,* lamprey *Petromyzon marinus*	4

## 2. Methods

The estuarine community has been sampled monthly for three decades. Fish samples are collected from the cooling-water filter screens at Hinkley Point ‘B’ power station, on the southern bank of the Bristol Channel in Somerset, UK (51°14′14.05″ N, 3° 8′49.71″ W). The water intakes are in front of a rocky promontory within Bridgwater Bay, while to the east are the 40 km^2^ Steart mud flats. Depending upon the tide, the fish are sampled from water varying in depth from about 8 to 18 m. A full description of the intake configuration and sampling methodology is given in Henderson and co-workers [14,15]. Methodology has not changed over the 30 years of study.

Quantitative sampling commenced in 1980 when 24 h surveys of the diurnal pattern of capture were undertaken in October and November. From these surveys, it was concluded that samples collected during daylight were representative of the 24 h catch, and monthly quantitative sampling commenced in January 1981. The total volume of water sampled per month, which has not varied over the 30-year period, is 4.27 × 10^5^ m^3^. To standardize for tidal influence, all sampling dates are chosen for tides halfway between springs and neaps, with sampling commencing at high water (normally about 12.00 h). The number and species of fish and crustaceans collected hourly from two filter screens over a 6-h period are recorded. Monthly samples are taken over 6 h on an intermediate tide in the spring–neap cycle because the rate of capture of many animals varies with the tidal height, and a standardized sample covering the average tidal range is considered most suitable when calculating annual rates of capture. Fortunately, this sampling regime works well for most species and gives adequate sample sizes for even low abundance species.

The power station intakes at Hinkley Point are an effective sampler because of their location at the edge of a large intertidal mudflat in an estuary with extremely powerful tides, which generate suspended solid levels of up to 3 g l^−1^, so that little light penetrates below 50 cm depth. Both pelagic and benthic fish are moved towards the intake in the tidal stream, often as they retreat from the intertidal zone where they feed. It is likely that they are unable to see or otherwise detect the intake until they are too close to make an escape. Light is clearly important for avoidance because captures are higher at night at power station intakes situated in clear water. The efficiency of the sampling method is discussed in Henderson & Holmes [14]. The filter screens have a solid square mesh of 10 mm and retain few fish less than 40 mm in length.

The wet weight of fish has been measured since 2000. This information was used in conjunction with data on numerical abundance to estimate the cumulative population biomass (i.e. biomass (in grams) summed over the duration of the survey) and the average body size (wet weight in grams) of individual species.

Data analyses used R [16]. The R package Kendall [17] was used to calculate Seasonal Mann Kendall tests, which enabled us to examine the consistency of guild rank order through time. To assess how the currency used to measure abundance affects our perception of guild capacity, we used a two-way ANOVA (currency × guild), repeated through years, in which guild size received a rank transformation [18].

## 3. Results

**Figure 1.**
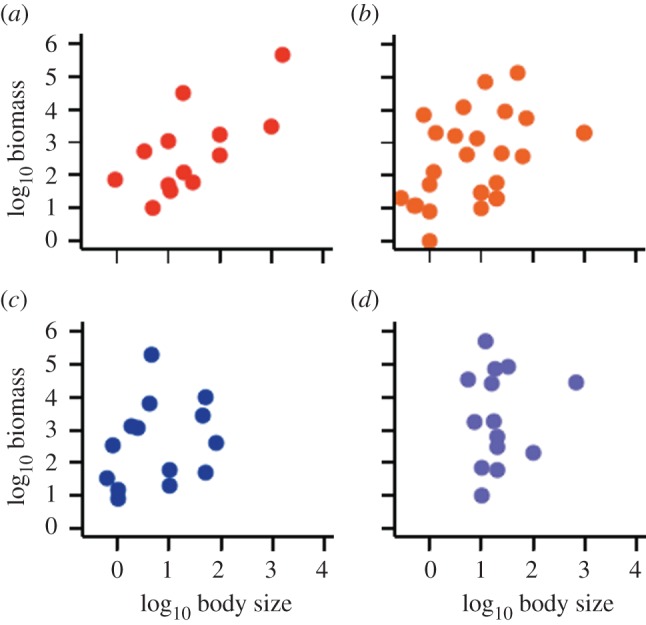
Relationship between abundance (biomass) and body size at the guild level. (*a*) hard-benthic, (*b*) soft-benthic, (*c*) pelagic, and (*d*) proximo-benthic guilds.

As expected, in the two spatial guilds that occur in habitats with substantial cover—the hard-benthic and soft-benthic guilds—larger bodied fish account for significantly more biomass (figure 1: hard-benthic *r*_s_ = 0.55, *p* = 0.04; soft-benthic *r*_s_ = 0.46, *p* = 0.01). In contrast, and as predicted, this relationship breaks down in the open habitats where fish will be most exposed to predators (figure 1: pelagic *r*_s_ = 0.37, *p* = 0.19; proximo benthic *r*_s_ = 0.02, *p* = 0.93; see also electronic supplementary material, figures S2 and S3).

**Figure 2.**
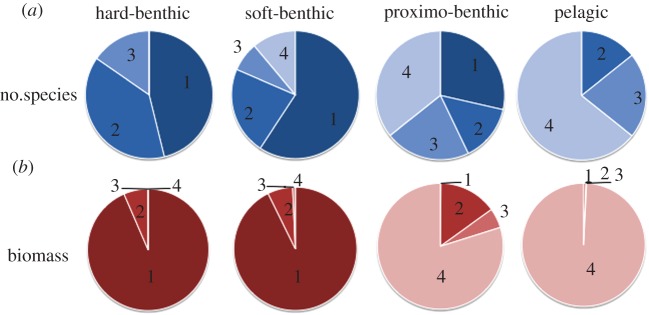
Fraction of (*a*) species richness and (*b*) biomass accounted for by species in the grouping categories across the four guilds depicted in figure 1. Species were categorized as follows: 1, mostly solitary; 2, occasionally in groups; 3, shoaling; 4, obligate schooling.

Fish species differ in the degree to which they associate in social groups [19] and range from solitary species, such as the conger eel (*Conger conger*) to obligate schooling species such as herring (*Clupea harengus*). To test our argument that shoaling is more frequent in open habitats, species were assigned to four categories—primarily solitary, occasionally found in groups, often shoaling and strongly schooling species—using [20,21]. An RxC *G*-test confirms that the frequency of species in each category varies across the spatial guilds *G* = 33.8 d.f. = 9, *p* < 0.001: strongly schooling species are common in the pelagic guild, less frequent in the proximo-benthic and soft-benthic guilds, and absent from the hard-benthic guild (figure 2*a*). Biomass has an even more striking allocation. Over 99 per cent of total biomass is associated with strongly schooling fish in the pelagic guild while greater than 90 per cent of biomass is contributed by primarily solitary species in the hard-benthic and soft-benthic guilds (figure 2*b*).

**Figure 3.**
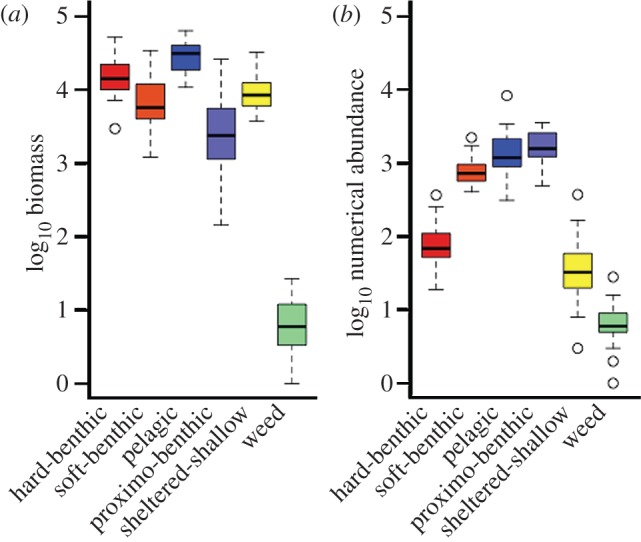
Guild capacity measured as (*a*) log_10_ biomass, and (*b*) log_10_ numerical abundance. Box plots show median value (across years) per guild, along with interquartile range, range and outlier values.

**Figure 4.**
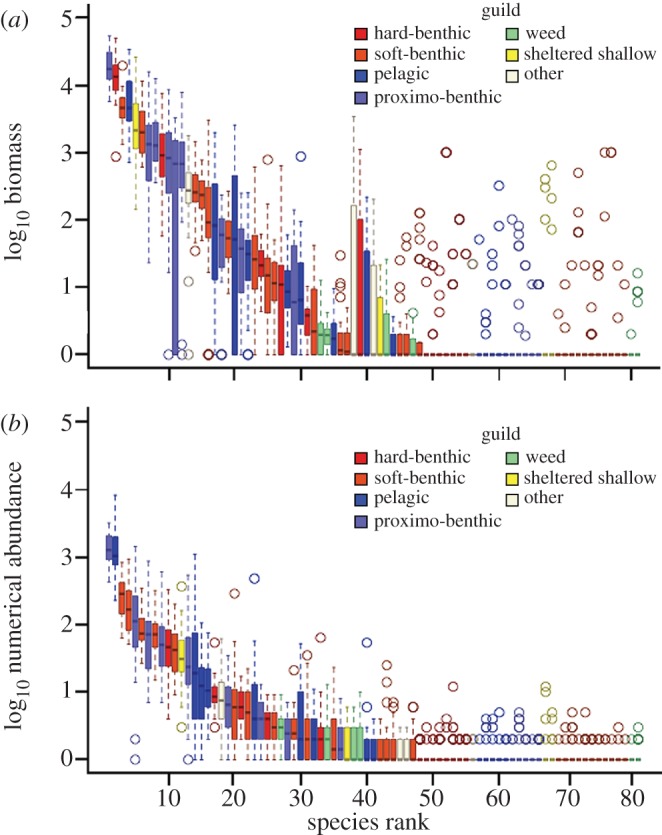
Rank abundance plots of (*a*) biomass and (*b*) numerical abundance. Box plots show median, interquartile range, range and outlier values for each species over the three decades of the survey. Species are ranked by (first) median and (second) upper quartile. Guilds are colour coded.

There is temporal turnover in species abundance and identity [22,23] with all guilds containing both core (species present in the majority of years) and occasional taxa (see electronic supplementary material, figures S4 and S5). The rank order of these guilds is however maintained through time, revealing that the fundamental structure of the community is conserved. This holds whether capacity is measured as biomass or as numerical abundance (Seasonal Mann Kendall test of guild rank order through time (years): biomass: τ = 0.09, *p* = 0.06; numerical abundance: τ = 0.063, *p* = 0.21). However, as a consequence of selection on body size, the relative position of the guilds in the assemblage changes when capacity is expressed in terms of numerical abundance (*F*_5,336_ = 20.07, *p* < 0.001). For example, the hard-benthic guild appears to have a low capacity if it is viewed in terms of the number of individuals it supports, but not in relation to its biomass (figure 3). Figure 4 shows how the overall SAD is produced when these guilds are overlain. It is notable that the five most abundant species in the distribution of biomass, which together contribute 76 per cent of overall biomass, belong to different guilds.

## 4. Discussion

These results demonstrate that ecological communities are composed of guilds that exploit available habitat in different ways and that follow different rules in how resources are divided among species. Because selection on body size varies across guilds, the rank of these guilds will shift depending on whether abundance is measured as biomass or as numerical abundance. We argue that the processes structuring the community cannot be inferred from the distribution of numerical abundance alone and that information on biomass is needed to explain resource allocation among species. This also means that SADs are the product of the processes that structure the individual guilds. There will be common and rare species in each of these guilds, but the relative abundance of these species will be shaped by the way selection operates in different habitats.

It is notable that the structure of the community is conserved through time, against the backdrop of temporal turnover in species abundance and identity. Previously, we have shown that species can be divided into core and occasional taxa [22]. Our new analysis reveals that there are core taxa (i.e. those present in the majority of years in the record) in all guilds (see figure 4 and electronic supplementary material, figures S4 and S5). This suggests that a few persistently dominant species exploit much of the available resource with transient species arriving in relatively small numbers on a stochastic basis or in response to temporary environmental changes, such as colder winters, the state of the North Atlantic oscillation or an increase in river flow due to higher rainfall.

In this analysis, we have focused on predation as an important selection pressure on body size. However, habitat structure will constrain selection in other ways. For example, the sizes of the interstitial pores in the different benthic zones will influence the sizes of the organisms that can live there. In addition, body size may be affected by a range of other factors, including pathogens, mating system and, where relevant, trophic level [4]. Moreover, while our arguments about selection on body size, and the likely consequences of this for the relationship with abundance, have been articulated in the context of this estuarine assemblage, other systems will also be composed of spatial guilds that experience different selection pressures. For example, Southwood *et al.* [24] tracked changes in a heteropteran community over 67 years and divided species into five groups associated with different habitats: water, herbage, trees, grasses and annuals. Similarly, specialist herbivores could be assigned to a guild living on a single species such as oak, or even found in a particular habitat found there, such as leaves [25]. Assemblages can also be deconstructed in other ways, such as on the basis growth form or life-history traits [26–28] and these factors will contribute to variation in species abundances.

Our results demonstrate that ecological communities comprise multiple functional groupings, but which differ in predictable ways in how available capacity is allocated among species. In doing so, they emphasize our need to take account of selection when interpreting SADs [29], and highlight the essential role that long-term-replicated ecological data play in understanding the structure of ecological communities. For instance, the insight that there is heterogeneity among guilds in how biomass is divided into individuals, offers a way of reconciling niche theory and neutral theory [30] and provides a testable hypothesis to explain why some SADs are multi-modal [31].
